# Overcoming the thermal regime for the electric-field driven Mott transition in vanadium sesquioxide

**DOI:** 10.1038/s41467-019-09137-6

**Published:** 2019-03-11

**Authors:** Flavio Giorgianni, Joe Sakai, Stefano Lupi

**Affiliations:** 1 Laboratory for Non-linear Optics, Paul Scherrer Institute, 5232 Villigen, Switzerland; 2 GREMAN, UMR 7347 CNRS and Université François Rabelais de Tours, Parc de Grandmont, 37200 Tours, France; 3 INFN and Department of Physics, University of Rome La Sapienza, P.Le A. Moro 2, 00185 Rome, Italy

## Abstract

The complex interplay among electronic, magnetic and lattice degrees of freedom in Mott-Hubbard materials leads to different types of insulator-to-metal transitions (IMT) which can be triggered by temperature, pressure, light irradiation and electric field. However, several questions remain open concerning the quantum or thermal nature of electric field-driven transition process. Here, using intense terahertz pulses, we reveal the emergence of an instantaneous purely-electronic IMT in the Mott-Hubbard vanadium sequioxide (V_2_O_3_) prototype material. While fast electronics allow thermal-driven transition involving Joule heating, which takes place after tens of picoseconds, terahertz electric field is able to induce a sub-picosecond electronic switching. We provide a comprehensive study of the THz induced Mott transition, showing a crossover from a fast quantum dynamics to a slower thermal dissipative evolution for increasing temperature. Strong-field terahertz-driven electronic transition paves the way to ultrafast electronic switches and high-harmonic generation in correlated systems.

## Abstract (web summary)

Thermal effects limit the speed of the electrically driven insulator-metal transition in V_2_O_3_ to tens of picoseconds. Here the authors show that under an intense THz-electric-field excitation the thermal regime can be overcome, achieving a purely electronic transition on ultrafast timescales.

## Introduction

In the electronic breakdown, conductive states are generated in an otherwise insulating phase by quantum tunneling of valence electrons across the band gap^1^. Electric field-driven tunneling, which occurs without any direct interaction with the lattice, can induce a purely electronic instantaneous transition of an insulator to a metal. This approach, which enables a purely electronic switching, is expected to shed light on the quantum nature and the role of the electronic interaction in strongly correlated systems^2–4^.

In Mott–Hubbard materials, the delicate interplay of electron, spin and lattice degrees of freedom can lead to exotic physical phenomena as Mott insulator-to-metal transition (IMT)^5,6^ and high-*T*_*c*_ superconductivity^7^. The Mott IMT is often accompanied by a reconfiguration of the lattice which cooperates with the electronic correlation during the phase transition^8^. Thus, the electronic correlation and structural interaction could not so far be disentangled as purely electronic Mott transition is hindered by Joule heating^9–11^. Recently, IMT transition by impulsive breakdown has been observed in *κ*-(ET)_2_Cu[N(CN)]_2_Br and VO_2_^12–15^. However, the role of the temperature, the crossover between thermal and electronic processes and the possibility to induce an ultrafast electric field-induced transition in a Mott–Hubbard system are completely unexplored.

V_2_O_3_, belonging to the vanadium oxide Magnéli phase^16^, is universally considered as a textbook example of the Mott–Hubbard physics. It undergoes a first-order IMT at *Θ*_IMT_ ~150 K from an antiferromagnetic insulating phase to a metallic phase associated with a monoclinic-to-corundum lattice transition. While the exact role of the Mott–Hubbard mechanism in many strongly correlated systems, as for VO_2_, remains hotly debated due to the not negligible Peierls distortion, the dominant role of the Coulomb repulsion forces between the electrons in opening the insulating gap in V_2_O_3_ is well established^17–20^. Thus, V_2_O_3_ can be assumed as a canonical system to study the THz electric field-driven insulator-to-metal Mott transition in strongly correlated oxides^21^.

A number of recent experiments on Mott insulators, including Cr-doped V_2_O_3_, has pointed out the first-order nature of the electrically driven Mott transition^22–24^. These experiments have shown that, using static and pulsed (on sub-nanosecond timescale) electric fields, the electronic switching takes place under electro-thermal breakdown of the Mott phase involving Joule heating while the observation of a pure electronic mechanism demands large electric fields at very short timescales. So far, fast electronics experiments on V_2_O_3_ have demonstrated that sub-nanosecond pulses can only drive thermal IMT switching. In this regime, the dynamics of transition is dictated by the nucleation and percolative growth of the metallic phase^25^ where phonon propagation sets the speed limit of the switching to tens of picoseconds^26–28^, not allowing any conclusive evidence of a non-thermal electric field-driven IMT^29^.

Here, using high-intensity non-resonant THz pumping and tracking the ultrafast dynamics through a short-wave infrared (SWIR) probe, we show that the thermal regime of the IMT in the V_2_O_3_ Mott–Hubbard material can be overcome accessing a pure electronic switching process with a sub-picosecond temporal evolution. In time domain, the electric field transient by the carrier THz pulse occurs on a timescale shorter than percolative nucleation of the metallic phase (thermal regime of the IMT ~10–100 ps) but slower than the collective oscillations of the ions (optical phonon modes ~0.1 ps). In such a temporal window not accessible with fast electronics, the electric field stimulus is able to selectively trigger a lattice-decoupled electronic switching process by quantum tunneling at low temperature. Increasing the temperature, the temporal dynamics shows a crossover from a fast non-thermal regime to a slower dissipative evolution for temperature approaching the transition temperature *Θ*_IMT_.

## Results

### Terahertz field-driven insulator-to-metal transition in V_2_O_3_

**Fig. 1:**
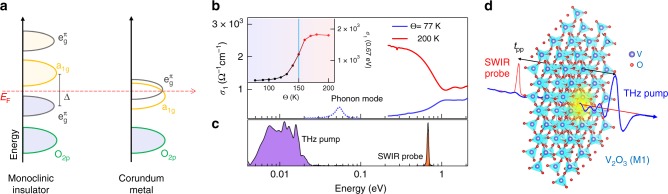
Electrodynamics properties of the V_2_O_3_ thin film and experimental set-up. **a** Energy level diagrams for the antiferromagnetic insulating (AFI) phase and paramagnetic metallic phase of V_2_O_3_. *E*_F_ denotes the Fermi energy and Δ represents the effective optical gap. **b** Real part of the optical conductivity of the V_2_O_3_ as a function of photon energy for 77 K (*Θ* < *Θ*_IMT_, insulating phase) and for 200 K (*Θ* > *Θ*_IMT_, metallic phase). Inset: Temperature dependence of the real part of the optical conductivity at the probe energy (0.67 eV). **c** Spectral distribution for the THz pump and short-wave infrared (SWIR) probe pulses. THz pump photon energy is below the lattice phonon modes and the interband transitions. SWIR probe energy measures the density of state at *E*_F_. **d** Schematic representation of the THz pump–SWIR probe experiment starting from the AFI monoclinic lattice structure of V_2_O_3_

We investigate a high-quality 82 nm thick film of V_2_O_3_ epitaxially grown on 500 μm-thick R-plane Al_2_O_3_ substrate by pulsed laser deposition technique (see Methods). Figure 1b shows the real part of the optical conductivity *σ*_1_(*ω*) at 77 K (<*Θ*_IMT_) and 200 K (>*Θ*_IMT_) as measured by Fourier transform infrared (FTIR) spectroscopy (see Supplementary Note 1 and Supplementary Fig. 3). The optical conductivity exhibits a clear IMT at *Θ*_IMT_ ~150 K upon heating (see the inset of Fig. 1b where the optical conductivity at 0.67 eV vs temperature is shown).

The strongly correlated nature of V_2_O_3_ leads to the formation of an antiferromagnetic insulator phase at low temperature (*Θ* < *Θ*_IMT_) where a large gap opens in the ${\mathrm{e}}_g^\pi$ states (Fig. 1a). Here, the effective optical gap Δ(~1.2 eV) is determined by the energy separation between the spin band, related to the ${\mathrm{e}}_g^\pi$ states below the Fermi energy, and the empty a_1*g*_ band^25^. In this phase the optical conductivity shows a maximum at ~1 eV (blue curve in Fig. 1b and Supplementary Fig. 3), which is related to the interband transitions between ${\mathrm{e}}_g^\pi$ and a_1*g*_. Across the IMT, the band gap closes leading to a non-zero density of states at the Fermi level (*E*_F_) through a prominent increment of the *a*_1*g*_ orbital occupancy. This results in a significant increase of spectral weight below 1 eV (red curve in Fig. 1b).

We use a non-resonant THz stimulus to trigger an ultrafast IMT in V_2_O_3_. The pump spectrum is strictly below the main optical phonon modes of V_2_O_3_ in the insulating phase (Fig. 1c), thus preventing a direct resonant excitation of the lattice^30^. Similar results are also achieved with a pump spectrum limited at 15.3 meV (see Supplementary Note 2 and Supplementary Fig. 4).

The electronic response to the THz excitation is probed in time using an ultrashort laser at photon energy of 0.67 eV. At this energy, as shown in Fig. 1c, the contribution to the optical conductivity of interband transition across Δ can be neglected due to the low joint density of states around the probe energy (see Supplementary Note 6). Thus, the optical response of the ultrashort SWIR probe is dominated by intraband transitions (see also Supplementary Figs. 7–9), and allows direct access to the metallic behavior^31^.

**Fig. 2:**
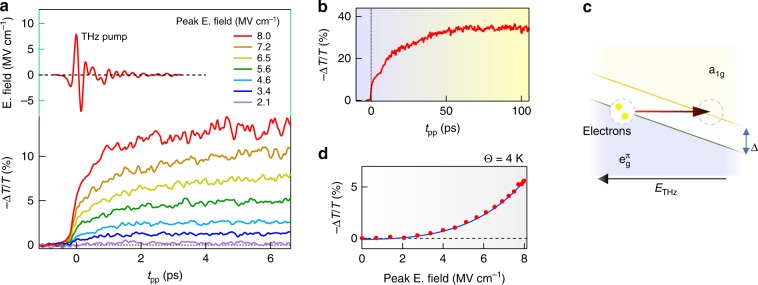
Terahertz-driven ultrafast electronic Mott transition in V_2_O_3_. **a** (top) Maximum THz pump field and (bottom) differential transmission modulation −Δ*T*/*T* at *Θ* = 4 K probed by short-wave infrared (SWIR) laser as function of different pump field strengths. The maximum peak *E*. field is 8.0 MV cm^−1^ (fluence, *F* = 16 mJ cm^−2^). **b** −Δ*T*/*T* On longer timescale at max *E*. field strength, **c** Non-resonant THz field induces energy band distortion and leads to an interband tunneling of electrons in a_1*g*_ resulting in a sub-ps Mott transition. **d** THz electric field strength dependence of −Δ*T*/*T* at *t*_*pp*_ = 0. The blue solid line corresponds to fit of −Δ*T*/*T*(*t*_*pp*_ = 0) for the electric field-assisted tunneling

Figure 1d shows the THz pump/SWIR probe scheme (see Supplementary Methods and Supplementary Fig. 1 for details). The temporal evolution of the THz-induced differential transmission (−Δ*T*/*T*) at the probe energy of the insulating phase of V_2_O_3_ (*Θ* = 4 K) as well as the single-cycle THz pump field are displayed in Fig. 2a. The time origin (*t*_*pp*_ = 0) is conventionally taken at the peak of the electric field. The temporal traces of −Δ*T*/*T* for field strengths between 2.1 and 8.0 MV cm^−1^ show a two-step dynamics characterized by a sudden suppression over the timescale of the THz pump excitation followed by a slow transient. The differential transmission turns constant after tens of ps (Fig. 2b). The rapid switching, over the sub-optical period of THz pump pulse (rise time, *t*_r_ < 0.3 ps), is the experimental fingerprint of a purely electronic transition^12^.

As the pump photon energy is far from the band gap (Δ/ℏ*ω*_pump_ ~30), direct multi-photon transitions give a negligible contribution, so the main effect of the strong THz electric field is to promote electrons into a_1*g*_ band by quantum tunneling. Indeed, due to the strong electric field, the a_1*g*_ and ${\mathrm{e}}_g^\pi$ bands start to be bended (Fig. 2c) and this induces the band crossing. The initial free carriers lead to a fast rise in the density of states at *E*_F_ and to a sudden metallization of insulating V_2_O_3_, as depicted in Fig. 2a. In this THz-induced tunneling process, the electric field dependence of the conductivity *σ*_1_(*E*) is proportional to exp(−*π*(*E*_th_/*E*)), where *E*_th_ is the THz electric field threshold. Indeed, −Δ*T*/*T* at *t*_*pp*_ = 0, reported in Fig. 2d, shows the typical trend of the tunneling process with a *E*_th_ = 6.7 MV cm^−1^ (see Methods). This exponential activation, which recalls the Landau–Zener quantum tunneling, characterizes the initial dynamics (*t*_*pp*_ = 0) in which the nucleation of conductive channels in the presence of a not yet collapsed Mott gap is exponential.

The THz-driven electronic tunneling, as discussed above, occurs in a sub-THz cycle (<0.3 ps) and ultimately triggers, in a slower timescale, the monoclinic-to-corundum lattice transition. Indeed, the energy deposited by the THz pulse through the electron–phonon thermalization (see Supplementary Note 4 and Supplementary Fig. 6) is sufficient to drive the thermal nucleation and percolative growth of the metallic phase^26–28,32^ generating additional conductive states, as by photo-excitation (see Supplementary Note 7 and Supplementary Fig. 10). The experimental fingerprint of these two sequential processes, THz-induced electronic tunneling and thermal nucleation, is mirrored in the two-step −Δ*T*/*T* temporal dynamics (see Fig. 2).

### Temperature evolution of the THz-driven IMT dynamics in V_2_O_3_

**Fig. 3:**
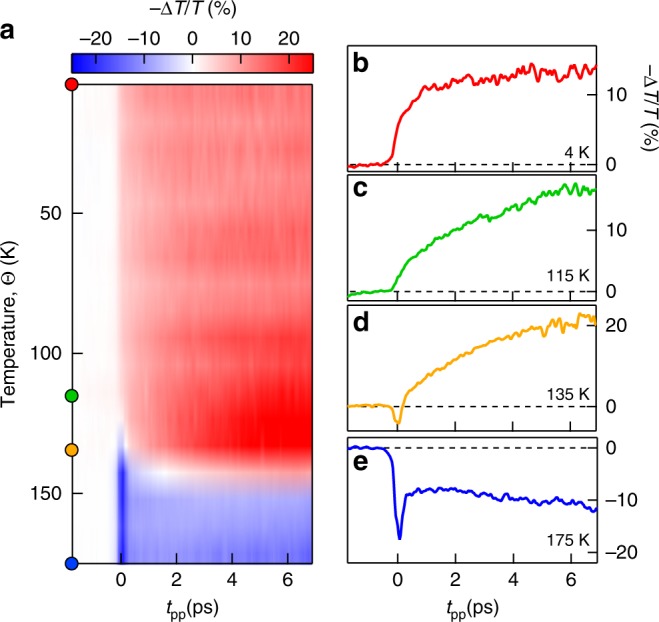
Temperature evolution of the pump–probe dynamics. **a** Temperature and time resolved −Δ*T*/*T* dynamics induced by the strong THz field. The temporal traces of the insulator-to-metal transition (IMT) −Δ*T*/*T* across the different regimes for background temperature of 4 K (**b**), 115 K (**c**), 135 K (**d**) and 175 K (**e**)

The THz-driven IMT largely changes as function of the background temperature. This variability indicates an interplay of quantum and thermal processes taking place during the initial temporal dynamics. Indeed, as shown in Fig. 3a, the dynamics of −Δ*T*/*T* changes sign and becomes negative by approaching *Θ*_IMT_ (see also Supplementary Note 8 and Supplementary Fig. 11). More specifically, the two-step IMT dynamics at Θ = 4 K driven by a THz electric field of 8.0 MV cm^−1^ (Fig. 3b) reduces to a slow single-step exponential trend already increasing the temperature to Θ =115 K (far below ΘIMT), as shown in Fig. 3c, −Δ*T*/*T* has a first negative variation followed by a slow positive single exponential increase. Above *Θ*_IMT_ (*Θ* = 175 K), when the thermal transition is complete, the dynamics turns again in a multi-step function constituted by a fast increase in transmission followed by a long-lived dynamics as observed in Fig. 3e. A similar behavior can also be observed at lower values of the THz field.

We first discuss the origin of the single-step dynamics in proximity of the transition temperature (*Θ* = 115 K), where the ultrafast tunneling breakdown regime is replaced by a slowly rising, long-lived metallization process. This behavior indicates a different interaction process which drives the phase transition. For temperature approaching *Θ*_IMT_, strongly correlated conducting states emerge through the formation of nanometric metallic puddles that fragment the insulating phase^18,33^. In this regime, the thermal processes due to the THz-driven leakage currents prevail over the electronic tunneling. The absorbed THz energy, which is proportional to the real part of conductivity *σ*_1_ according to the Joule heating model: ${\int} {\sigma _1} E(t)^2dt$, where *E*(*t*) is the THz pump field temporal profile, increases by 3 orders of magnitude from 4 K to 125 K due to the increase of conductivity (*σ*_1_(125 K)/*σ*_1_(4 K)~10^3^). The Mott system is then driven into a dissipative regime by the THz pulses when the absorbed energy overcome the latent heat associated to the first-order structural phase transition^26^. In this regime, the dynamics is dominated by nucleation and percolation to the metallic corundum phase giving rise to a slower evolution (*t*_r_ ~30 ps) compared to electron–phonon thermalization (see Supplementary Note 3 and Supplementary Fig. 5).

At *Θ* = 175 K, above the thermal IMT temperature, a fast dynamics comparable to the pump stimulus duration is associated to an increase of the optical probe transmission (negative −Δ*T*/*T*). This fast transient, which reflects the absence of a percolative kinetics, is related to the generation of hot carriers by interaction with the THz pulse. Indeed, rapid reduction of absorption can be ascribed to intervalley and intervalley scattering dynamics of the hot electrons as observed in doped semiconductors^34–36^.

The energy deposited by the THz pulse leads to an increase of the electronic temperature. Indeed, the magnitude of the drop in −Δ*T*/*T* (after the fast transient) is consistent with the heating process which causes an estimated temperature increase of ~200 K (see Supplementary Note 5). Since the metallic phase of V_2_O_3_ is governed by electronic correlations, for temperature approaching the coherence temperature *T*_ch_ ~400 K, the lifetime of the quasiparticles is reduced in both a_1*g*_ and ${\mathrm{e}}_g^\pi$ bands, weakening the metallic response^30^.

**Fig. 4:**
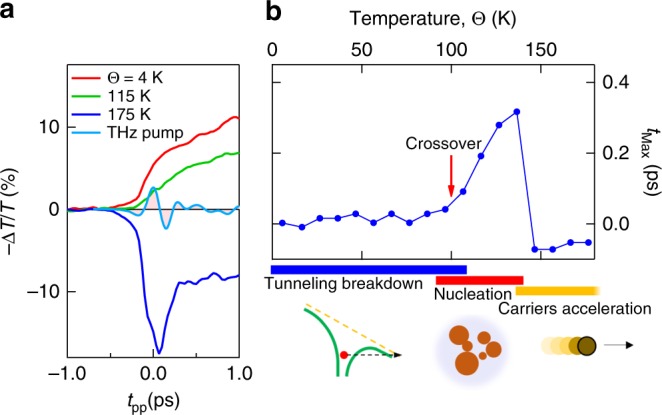
Crossover from purely electronic to thermal regimes of THz-driven insulator-to-metal transition dynamics in V_2_O_3_. **a** Temporal dynamics of −Δ*T*/*T* at different temperatures induced by THz electric field of 8.0 MV cm^−1^ (light blue curve). **b** Temperature evolution of the time value *t*_max_ of the maximum of the derivative of −Δ*T*/*T*. The temperature turns the THz-driven Mott transition across three different regimes: tunneling breakdown at low temperature; nucleation and growth of metallic domain due to Joule heating in the presence of conductive states; carriers accelerated by THz pulse leading to the ultrafast reduction of the plasma frequency

The rich variability of THz interaction in Mott systems as function of temperature can also be visualized in the characteristics transition time of −Δ*T*/*T*. Figure 4a displays the initial dynamics (limited to 1 ps), of −Δ*T*/*T* vs. temperature. The light blue curve shows the profile of the THz pump electric field. The initial dynamics −Δ*T*/*T* for the insulating phase (*Θ* <*Θ*_IMT_) and metallic phase (*Θ* > *Θ*_IMT_) mimic the THz electric field with a sub-cycle rise time. In contrast, the dynamics for *Θ* = 115 K is delayed being the temporal evolution dictated by the nucleation and growth of the metallic phase (thermal regime). At 175 K (*Θ* > *Θ*_IMT_), when the temperature-induced transition to the metallic phase is completed, the dynamics change sign. Here, the ponderomotive electric field accelerates the free carriers, causing a sudden increase of the optical transmission (see above).

To better capture these features, in Fig. 4b we report the temporal maximum (*t*_max_) of the derivative of −Δ*T*/*T* vs. temperature. From 4 K, the time characteristics remains constant until 100 K; in this range of temperature, the switching is driven by purely electronic quantum breakdown. At higher temperature, *t*_max_ increases because the dynamics enters in a dissipative regime where the THz-induced leakage current leads to slow nucleation. Above 140 K, *t*_max_ drops again due to the instantaneous electronic response of the metallic phase. In the crossover regions a combined dynamics is observed.

### Mechanical stress in metallic V_2_O_3_ induced by THz fields

**Fig. 5:**
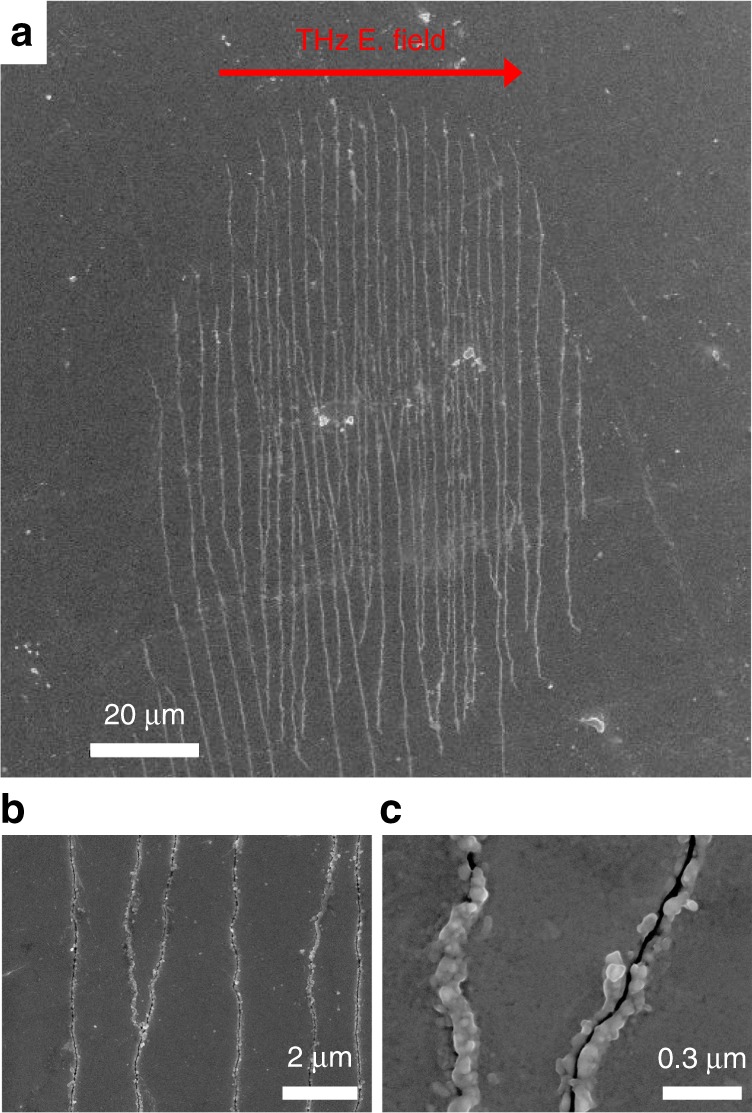
Critical fractures in metallic V_2_O_3_ thin film induced by intense THz pulses. Scanning electron microscopy (SEM) images of **a**–**c** microcracks in the metallic V_2_O_3_ thin film (*T* = 300 K) induced by THz pulses with an electric field of 12.7 MV cm^−1^ (*F* = 37 mJ cm^−2^). **a** Micrometric scale image of the whole damage region. THz beam profile: 180 μm (1/e^2^ width). Scale bar is 20 μm. The cracks have a periodicity of ~2 μm (**b**) and a width of ~20 nm (**c**). Thin-film fracture is only observed in the metallic phase. Scale bars in **b** and **c** are respectively 2 μm and 0.3 μm

Another remarkable aspect of THz-induced carrier acceleration in the metallic phase of V_2_O_3_ is that a large ponderomotive force acting on the carriers along the THz electric field direction results in a critical stress parallel to the film surface. Indeed, for a THz pulse with an electric field strength of 12.7 MV cm^−1^ (fluence, *F* = 37 mJ cm^−2^), the THz-induced impulsive stress is strong enough to generate mechanical fractures on the film surface, as shown in Fig. 5. The observed damage resembles an array of microcracks elongated perpendicular to the THz electric field direction. Additional scanning electron microscopy (SEM) images are reported in Supplementary Fig. 2. Both the micrometric periodicity and the nanometric width of the cracks are not related to the THz pump wavelength. This electrostriction effect has been recently observed in metal thin films irradiated by intense THz pulses below the ablation damage threshold^37^. Since the tensile force on the film applied by the THz field is proportional to the optical conductivity, in the insulating phase of V_2_O_3_ we do not observe any sample damage for the electric field strength of 12.7 MV cm^−1^.

## Discussion

We report an ultrafast insulator-to-metal transition in the archetypal Mott–Hubbard system V_2_O_3_. The purely electronic IMT is achieved by quantum tunneling under the effect of atomically strong THz electric field. This excitation allows to access new metallic precursor phases in Mott systems by tunneling breakdown, overcoming the transition speed limit imposed by the thermal IMT accessible by fast electronics.

Our approach reveals the interplay of the thermal and electronic switching processes. The electric field-driven Mott transition shows that the quantum tunneling regime can be disentangled from the Joule heating with the temperature. Indeed, when the background temperature is well below the *Θ*_IMT_, a pure tunneling breakdown leads to the metal monoclinic precursor phase on a sub-ps timescale followed by a subsequent lattice rearrangement. Close to the insulator-to-metal transition temperature, the IMT takes place in tens of picoseconds due to the predominance of Joule heating by direct interaction of the THz field with the conductive states. In the metallic phase, above *Θ*_IMT_, the free carriers are accelerated by the THz ponderomotive field leading to a sudden increase of optical transmission. This ultrafast interaction between the THz generated hot carriers and the host crystal lattice drives impulsive strain waves on metallic V_2_O_3_. In fact, for an electric field of 12.7 MV cm^−1^, THz-induced critical shear stress mediated by carrier acceleration results in cracking of the V_2_O_3_ thin film.

The THz-driven IMT studied here opens promising pathways towards faster and more efficient switches in correlated oxides for the development of new Mottronics devices and in laser technology in terms of high-order harmonic generation^38–40^.

## Methods

### Sample characterization and growth

Growth technique, diffraction analysis and transport measurements for the sample used in the experiment are reported in ref. ^41^. Specifically the sample (82 nm thin film of polycrystalline V_2_O_3_) has been grown on R-plane sapphire substrate by pulsed laser deposition with a fluence of 1 J cm^−2^.

### Transmission modulation induced by terahertz fields

The two-step behavior of the transmission modulation in Fig. 2a can be explained by a THz-driven increase of the optical conductivity by interband tunneling mechanism.

The electric field-dependent intraband optical conductivity is given by^4,13^

1 $$\sigma _1(E) = \sigma _\infty {\mathrm{exp}}( - \pi E_{{\mathrm{th}}}/E),$$

where *σ*_∞_ and *E*_th_ are the optical conductivity and the electric field tunneling threshold. Combining this equation with the Tinkham formula (see Supplementary Note 1), we fit the differential transmission Δ*T*(*E*) = (*T*(*E*) − *T*_0_)/*T*_0_ as a function of pump electric field strength *E*, where *T*_0_ is the transmission at 1.8 μm without the THz pump with respect to the bare substrate. As shown in Fig. 2d of the main manuscript, the fitting curve reproduces very well the experimental data obtaining *E*_th_ = 6.7 MV cm^−1^ and *σ*_∞_ = 820 cm^−1^ (which is compatible with the value of Drude component of the optical conductivity at the probe frequency) with *T*_0_ fixed at 0.51 (which corresponds to the transmittance measured in the insulating phase at the probe wavelength). This electric field threshold corresponds to a correlation length *ξ* = Δ/2*E*_th_^4^ of ~0.7 nm, which is close to the estimated value for V_2_O_3_^9^.

## Supplementary information

Supplementary Information

Source Data

## Data Availability

The source data underlying Figs. 1b, c, 2a, b, 3b–e, 4b and Supplementary Figs. 1b, 3a, b, 4a, b, 5–7, 9, 10a, b, 11b. are provided as Source Data file. SEM images and other findings of this study are available from the corresponding author upon reasonable request.
