# Supplementary material for: Overcoming the thermal regime for the electric-field driven Mott transition in vanadium sesquioxide
Source: Nat Commun. 2019 Mar 11;10:1159. doi: 10.1038/s41467-019-09137-6 (PMC6411733; doi:10.1038/s41467-019-09137-6)
Supplement: Supplementary file 1 — Supplementary Information [file 41467_2019_9137_MOESM1_ESM.pdf]

Supplementary Information for:

Overcoming the thermal regime for the electric-field driven Mott transition in vanadium sesquioxide

Flavio Giorgianni[1], Joe Sakai[2] and Stefano Lupi[3]

[1]*Paul Scherrer Institute, Laboratory for Non-linear Optics, 5232 Villigen PSI, Switzerland*

[2]*GREMAN, UMR 7347 CNRS and Université François Rabelais de Tours, Parc de Grandmont, 37200 Tours, France*

[3]*INFN and Department of Physics, University of Rome La Sapienza, P.Le A. Moro 2, 00185 Rome, Italy*

Supplementary Methods. THz pump-SWIR probe setup

We use the output of a 100 Hz, 20 mJ, 45 fs Ti:Sapphire laser to drive an optical parametric amplifier (OPA) which provides both signal and idler beams. The signal spectrum is centered at 1.5 μm while the idler at 1.8 μm. The idler was splitted in two beams before the sample; the first beam was used to probe the transmission of the sample (SWIR probe) while the second one was measured by a reference detector to implement the differential detection scheme (see Supplementary Fig. 1**a**). The spot size of the probe beam on the sample is 68 μm ($1/e^2$ width).

Single-cycle THz pump pulses were generated from the OPA signal beam by optical rectification in DAST crystal[1]. Metal mesh low-pass filter, inserted into the optical path of the THz, was used to cut-off frequencies higher than 6 THz. The maximum THz energy for the experiment was E_{THz} =9.6 μJ.

THz beam was focused on the sample inside a cryostat (Montana Cryostation) by a parabolic mirror with a focal length of 3 inch. The beam focus measured by a THz camera (NEC Terahertz Imager) is shown in Supplementary Fig. 1**b** (blue curve). The spot size is 276 μm ($1/e^2$ width), which corresponds to a fluence onto the sample of 16 $mJcm^{-2}$. The THz pulse temporal profile measurement at the sample position was performed by electro-optic sampling (EOS) using a (110) GaP crystal with a thickness of 50 μm. The measured full-width half maximum THz pulse duration is τ_{FWHM}=0.166 ps. The electric field strength $E_{THz} = \sqrt{z_0 I_{THz}}$ =8.0 $MVcm^{-1}$ has been calculated from peak THz intensity[2]:

$$I_{THz} = \frac{4\sqrt{ln2}}{\pi\sqrt{\pi}} \frac{E_{THz}}{w^2 \tau_{FWHM}} \tag{1}$$

where w is the beam waist.

At this electric field strength (F=16 $mJcm^{-2}$), no damage has been observed on the V_2O_3 film for both insulating and metallic phase. High intensity THz measurements were carried out focusing the THz beam by means of a parabolic mirror with 2 inch focal length. The beam profile on the focus is shown in Supplementary Fig. 1**b** ($1/e^2$ width 180 μm), red curve, which corresponds to a fluence of 37 mJ/cm^2 and to a THz electric field strength of 12.7 $MVcm^{-1}$. At this electric field, in the metallic phase we observed micro-cracking damage due to THz driven electrostriction as discussed in the main manuscript. Additional SEM images of the micro-crack details in V_2O_3 thin film are reported in Supplementary Fig. 2.

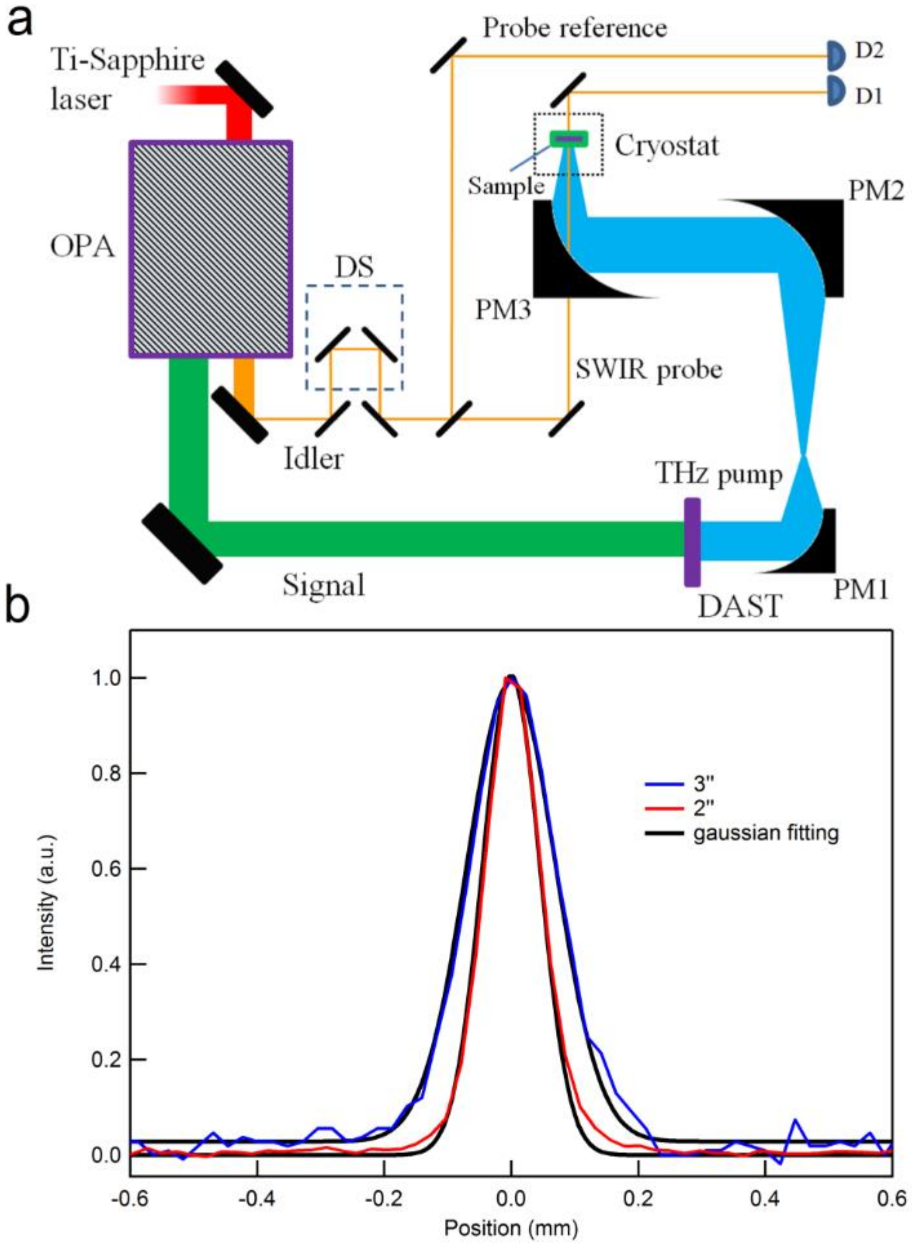

Supplementary Fig. 1: THz pump vs SWIR probe setup. a) THz pump-SWIR probe scheme: PM: parabolic mirror, DS: delay stage, OPA: optical parametric amplifier, D: detector. **b**) THz beam profiles on the sample focal plane using a parabolic mirror (PM3) with 2 inch (red curve) and 3 inch (blue curve) of focal length. Black curves represent a Gaussian fit. The $1/e^2$ widths are 180 μm and 276 μm, respectively.

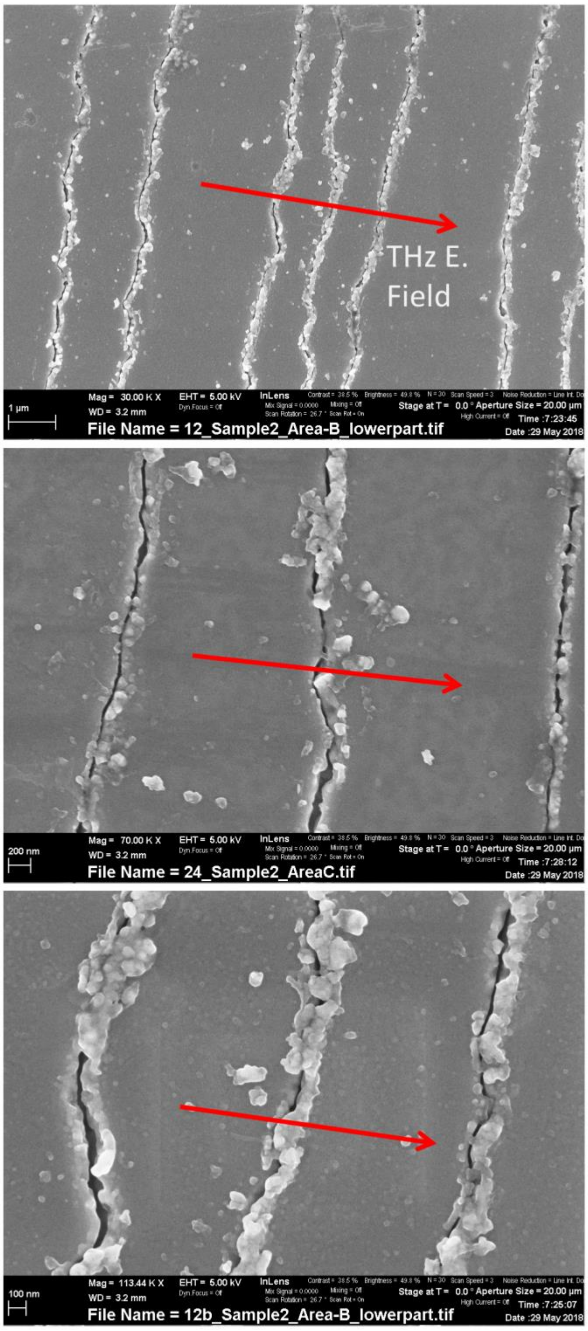

Supplementary Fig. 2: Critical strain and damage induced by THz pulse on metallic V_2O_3. SEM images of the micro-crack details in V_2O_3 thin film. The high current density driven by an intense THz pulse induces stress waves on the film surface propagating along the electric field direction (red arrows). For an electric field of 12.7 MV/cm, the critical shear stress results in a group of micro-cracks which follow the equipotential lines of the THz field. An accumulation of material is also observed at the edges of the cracks due to the strong mechanical stress. (Number of shots, N=10000).

Supplementary Note 1. Optical conductivity of V_2O_3 thin film in steady state regime

The temperature evolution of the electrodynamics properties in the mid-infrared to optical region across the insulator-to-metal transition of the V_2O_3 film was characterized by measuring the optical

transmittance using Fourier transform infrared (FTIR) spectroscopy. The real part of the optical conductivity σ_1 vs frequency, depicted in Supplementary Fig. 3**a**, is calculated by the Tinkham formula[3,4]
$T = 1/(1 + z_0\tilde{\sigma}d/(n + 1))^2$, where $z_0 = 377\ \Omega$ is the vacuum impedance, d is the thickness of the V_2O_3 film, n is the refractive index of the substrate and $\tilde{\sigma}$ the complex optical conductivity of the V_2O_3. For the V_2O_3, in our spectral range the contribution of the imaginary part of $\tilde{\sigma}$ to the transmittance is negligible, therefore is reasonably to assume $T = 1/(1 + z_0\sigma_1 d/(n + 1))^2$.
The insulating monoclinic phase ($\Theta < \Theta_{IMT} = 150\ K$) shows an energy gap > 0.6 eV[5] approximately related to the transition between e_g^{π} and a_{1g} bands.
When the IMT is concluded, the metallic phase is characterized by a Drude-like lineshape in agreement with previous measurements[5]. The Tinkham formula is applicable if $\nu/cdn < 1$ (with ν frequency of light; c speed of light; d sample thickness; n refractive index of V_2O_3). In both metallic and insulating phase, this condition is always satisfied at the probe wavelength[6]. In Supplementary Fig.43 we compare the optical conductivity by Tinkham formula (panel **a**) with the one computed by the Kramers-Kronig (KK) constrained analysis (panel **b**) which provides the exact solution[7]. The good agreement of these two results further confirms the applicability of the Tinkham formula.

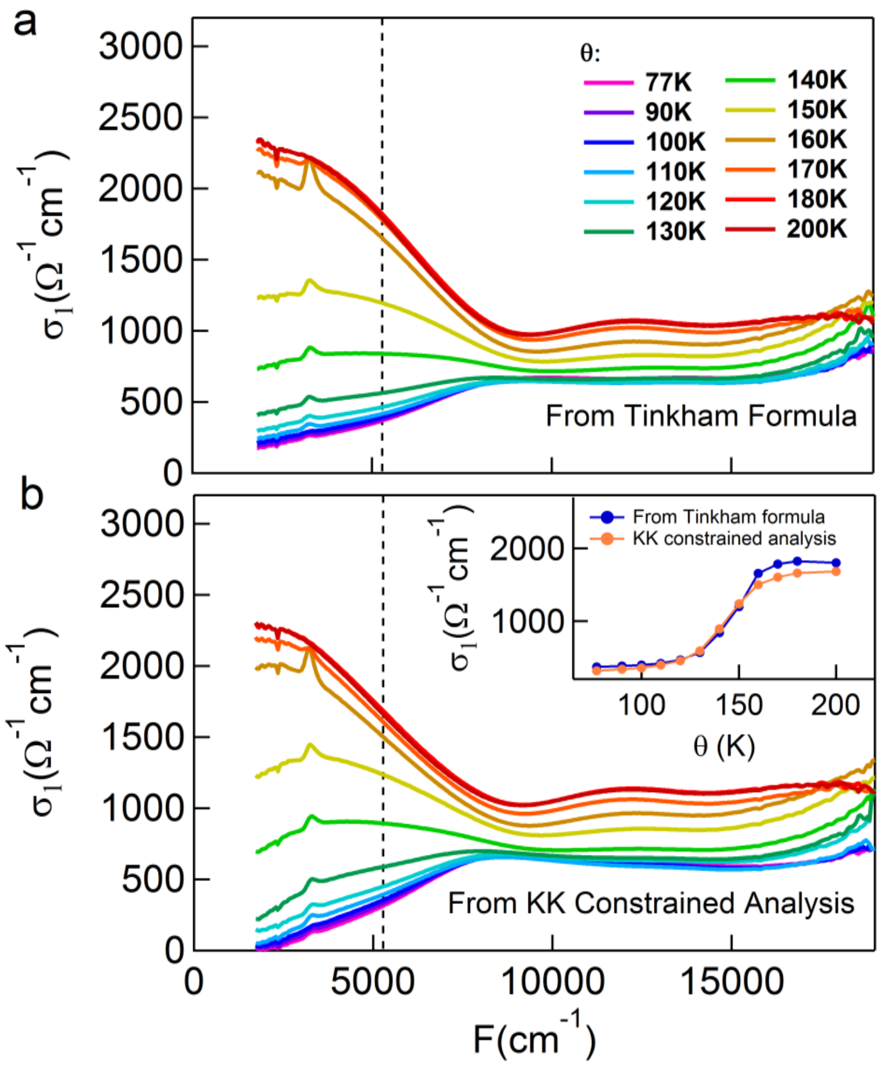

Supplementary Fig. 3: Temperature dependence of the real part of the optical conductivity calculated using the Tinkham formula (**a**) and Kramers-Kronig (KK) constrained analysis (**b**) from the transmittance measured by FTIR. Dashed line indicates the probe frequency. Inset: Comparison of the two methods at the probe wavelength. The small peak between 3200 cm^{-1} and 3700 cm^{-1} is from the not perfect compensation of

the transmission measurements. It is related to the O-H stretching mode of the water because the FTIR measured were carried out with the cryostat at normal air conditions.

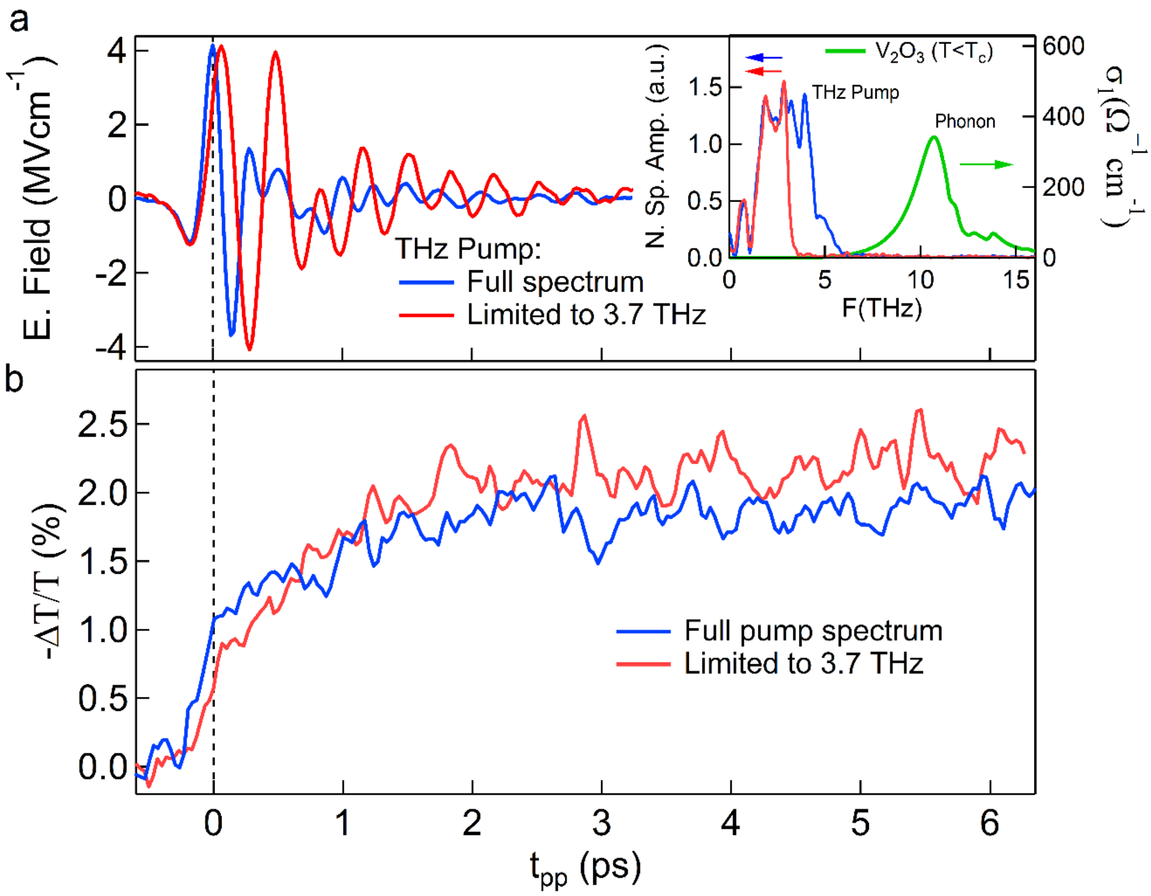

Supplementary Fig. 4 IMT dynamics *vs.* the pump spectrum. a, Electric field profile of the THz pump pulses: pump pulse with the full-band spectrum used in the main manuscript (blue curve) and spectrally limited to 3.7 THz. Inset: relative spectra which are far below the phonon band of V_2O_3 (green curve). The optical conductivity is related to bulk single crystal of V_2O_3[11]. **b**, Temporal evolution of transmission modulation at $\Theta = 4\ K$ driven by a THz pump with spectrum used in the main experiment (blue curve) and driven by a THz pump with a spectral cut-off at 3.7 THz (red curve). The THz electric field strength is ~4 MVcm^{-1}.

.

Supplementary Note 2. Ultrafast IMT by THz non-resonant pumping

As discussed in the main text, the terahertz field acts as off-resonant excitation with photon energy below the lattice phonons and the interband transitions. In order to exclude any possible resonant excitation with the lattice as mechanism to drive the transition, the transmission modulation of the SWIR probe was measured reducing the pump spectrum to 3.7 THz by means of a THz low-pass filter (see Supplementary Fig. 4**a**).

In Supplementary Fig. 4**b**, the temporal traces of -ΔT/T are reported for the two different THz pumps. As one can clearly observe, the pump-probe dynamics is comparable for the two THz pumps and it is appreciably independent of the spectral content of the pump (which are both below the phonon band). Thus we can conclude that the IMT is driven by a non-resonant THz excitation.

Finally, we can exclude a multi-photon process involving optical phonons in driving the IMT. As the latent heat of the V_2O_3 associated to the first order structural transition is 70 mJcm^{-3} (see Ref.8), for the illuminated sample volume in our experiment (thickness 82 nm, diameter 276 μm), we can estimate that an energy of 0.024 uJ is required to carry out the transition involving optical phonons. The THz pump energy in our experiment is 9.6 μJ. We can roughly estimate that the efficiency of the multi-photon excitation must be 10^{-3} to achieve the IMT.
In our knowledge, no multiphoton process of up-conversion (especially at THz frequency) has such conversion efficiency. This suggests that the increase of phonon population by multi-phonon processes does not play a role in the ultrafast IMT.

Supplementary Note 3. THz-induced nucleation dynamics

Close to the IMT temperature (at $\Theta = 115\,K$), the optical conductivity increases and the current generated by the THz pump prevents the tunnel mechanism. Due to the Joule heating, as discussed in the main text, the sub-ps insulator to metal switching is replaced by a slow thermal dynamics. The temporal evolution of -ΔT/T, associated to the nucleation and growth of the metallic domains, shows an exponential evolution with a rise-time of ~28 ps as shown in Supplementary Fig. 5.

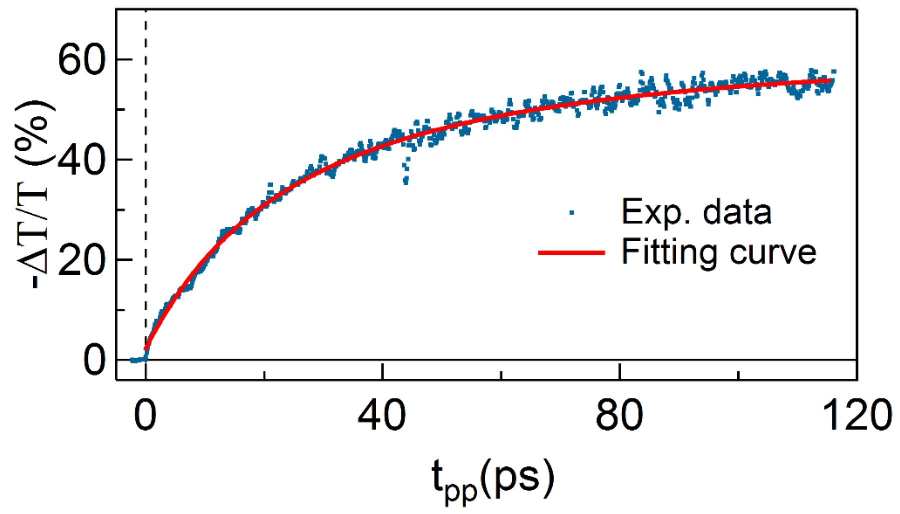

Supplementary Fig. 5: Nucleation dynamics of the IMT induced by a THz pulse. THz driven temporal dynamics of ΔT/T. The blue dots are experimental data and the red solid line fits to a single exponential. $\Theta = 115\,K$. The pump electric field is 8.0 MVcm^{-1}.

Supplementary Note 4. Nonlinear THz transmission of the V_2O_3 thin film at 4 K in the high field regime

Even at the minimum temperature (4 K), after a fast rise due to the tunneling processes the dynamics shows a slow increase related to the thermal nucleation of the metallic phase caused by THz induced electronic heating (see Fig. 2**a**-**b** in the main manuscript).

In order to estimate the dissipated THz energy we measure the THz transmission of the V_2O_3 film at 4 K as a function of the THz electric field strength (see setup in Supplementary Fig. 6).

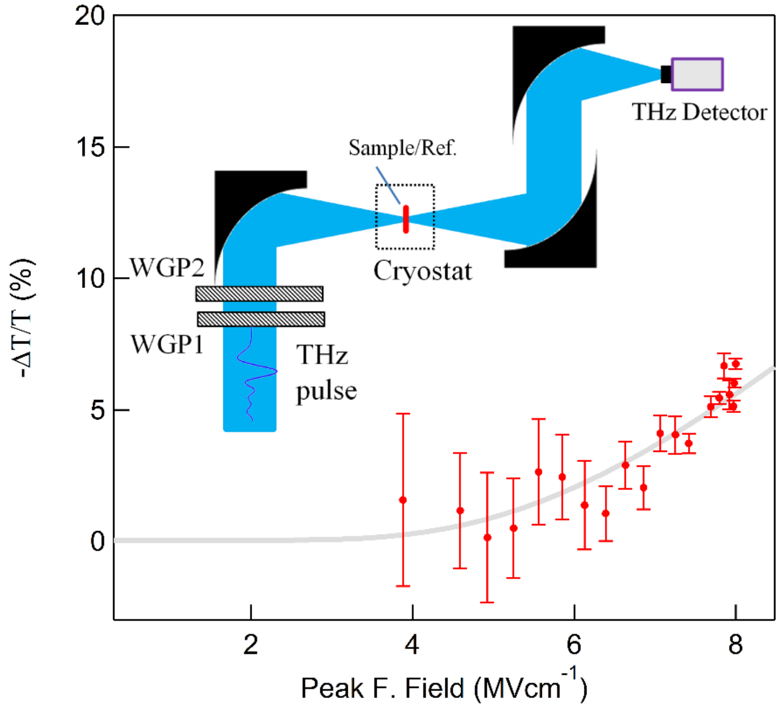

Supplementary Fig. 6: Nonlinear THz transmission modulation of the V_2O_3 thin film at 4 K *vs.* electric field amplitude: experimental setup and measurements. Error bars indicate the s.d. of the mean determined from 15 measurements.

Nonlinear THz transmission modulation *vs.* THz electric field was measured tuning the amplitude of the THz electric field with a pair of wiregrid polarizers (WGP1 and WGP2 in Supplementary Fig. 6). The transmitted intensity of the sample and reference was detected by a Golay cell (THz detector in Supplementary Fig. 6). We observed an exponential decrease of the THz transmission (increase of –ΔT/T) due to the THz driven metallization. At the maximum electric field, we observe a decrease of the transmittance of ~6%. By means of Reffit software[7], we estimate an optical absorption of ~5% which corresponds to a deposition on order of ~0.5 μJ of THz energy. This dissipated THz energy is comparable with the latent heat (0.34 μJ) associated to the first-order structural transition[8]. Therefore, it is realistic that, after the tunneling dynamics, the THz heating causes the thermal nucleation and the slow growth of the metallic phase as observed in Fig. 2**a**.

Supplementary Note 5. Increase in electronic temperature in metallic V_2O_3 by THz joule heating

In the metallic phase ($\Theta > 145\ K$), the THz pump pulse enhances the electron energy through Joule heating leading to an increase of the electronic temperature Te which results in an increase in optical transmission (–ΔT/T<0), as discussed in the main manuscript. Indeed, as V_2O_3 is a strongly correlated metal, the electronic correlations greatly renormalize the coherence temperature T_{ch}~ 400 K. The rise of the electronic temperature T_e, to T_{ch}, causes a strong reduction of the Drude spectral weight[9]. Here, we evaluate the increase of T_e by the THz energy density deposited in the sample and we show that it is compatible with the measured transmittance increase.

The parameters of our experiment are: THz pulse energy: 9.6 μJ; initial background temperature: 175 K; V_2O_3 transmittance in THz range ~ 10%; Spot size (diameter 1/e^2): 276 μm; Sample thickness: 82 nm; V_2O_3 electron heat capacity at C(T_e): γ T_e, with γ=80 mJK^{-2}mol^{-1} (Ref. 10); V_2O_3 density 4.87

gcm^{-3}. At 175 K, we estimated a fast increase of the electronic temperature by THz Joule heating in the metallic phase of ΔΘ~200 K. For this temperature enhancement, infrared measurements show a drop of the NIR optical conductivity of 100÷300 Ω^{-1}cm^{-1} (Ref. 11), which is consistent with a transmittance variation –ΔT/T of 10%.

Supplementary Note 6. THz pump SWIR probe Vs THz probe

In the insulating phase of V_2O_3, the optical gap Δ~0.6 eV is defined through the minimum energy difference between the a_{1g} and e_g^{π} bands. However, the small joint density of states of the interband transitions at 0.67 eV (photon energy of the SWIR probe), results in a negligible contribution to the optical conductivity (see Supplementary Fig. 7**a**). Indeed, the maximum of the interband contribution to the optical conductivity is located at ~1.2 eV (effective optical gap), far above the probe photon energy[5,12].

In the metallic phase, the optical response at the probe photon energy of 0.67 eV is mainly dominated by intraband transitions, which are related to the metallic behaviors. This behavior is described in Supplementary Fig. 7**b**, where we analyze the electrodynamics of paramagnetic metallic phase of V_2O_3 through a Drude-Lorentz multicomponent fit (see Ref. 13 for details). The red curve represents the Drude + MIR band (as discussed in Ref. 13) while the vertical dashed black line shows the probe frequency (5500 cm^{-1}). As one can observe, at the probe frequency, which is far below the Drude plasma frequency (ω_p~11000 cm^{-1}), the intraband contribution (metallic behavior) is the main contribution to the optical conductivity.

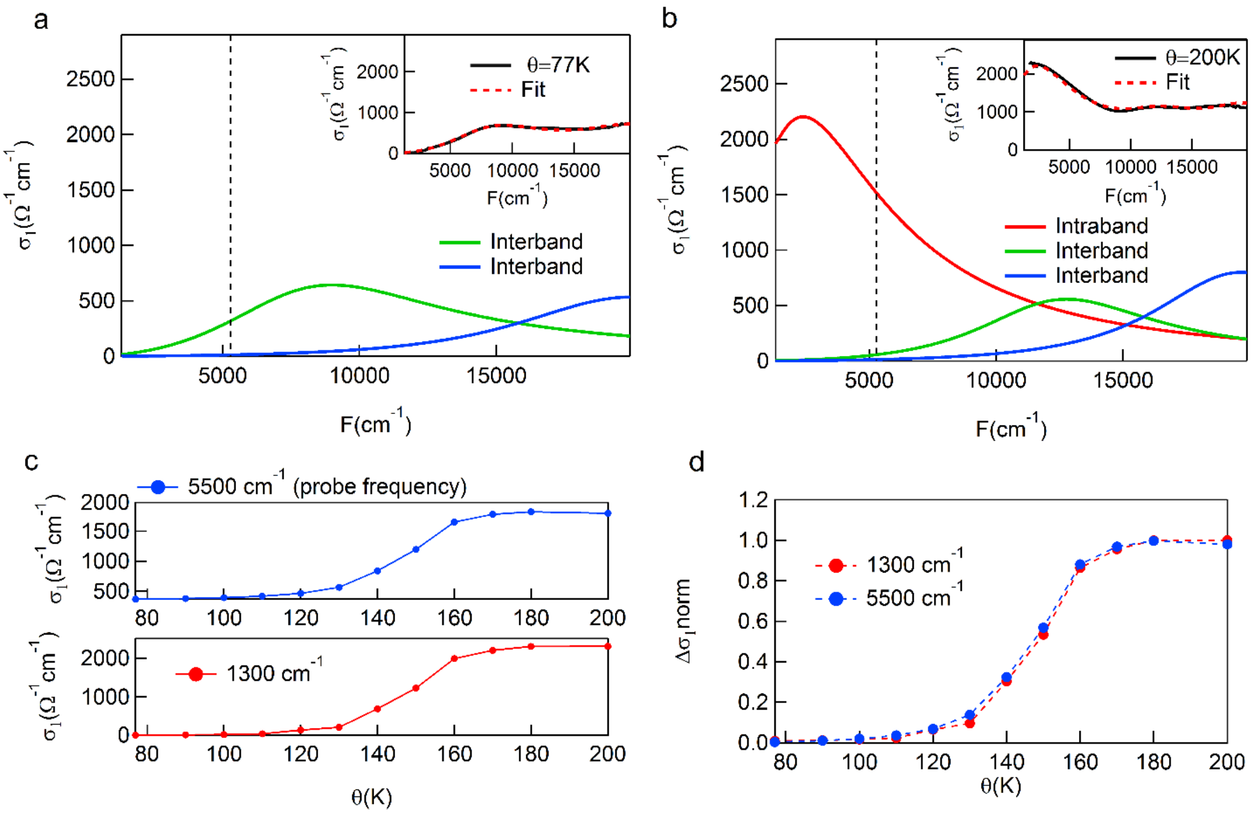

Supplementary Fig. 7. Real part of the optical conductivity σ_1 of V_2O_3 as a function of photon frequency and its analysis in terms of Drude-Lorentz components. a) Interband transition components (green and blue

curves), that contribute to $\boldsymbol{\sigma_1}$of the insulating phase (Θ=77 K). These components have been extracted through a multi-component Drude-Lorentz fit of $\boldsymbol{\sigma_1}$ (see inset). **b)** Interband transition components (green and blue curves), and metallic (Drude+MIR band) terms contributing to $\boldsymbol{\sigma_1}$ of the metallic phase (Θ=200 K). These components has been extracted through a multi-component Drude-Lorentz fit (see inset). **c)** Temperature dependence of $\boldsymbol{\sigma_1}$ at different photon frequency (5500 cm^{-1} and 1300 cm^{-1}). **d)** Normalized increment of $\boldsymbol{\sigma_1}$ vs temperature at 5500 cm^{-1} and 1300 cm^{-1}.

Moreover, in Supplementary Fig. 7**c**, we compare the temperature dependence of the optical conductivity at 5500 cm^{-1} (probe frequency) with the one at 1300 cm^{-1} (photon energy well below the optical gap 1300 cm^{-1}~39 THz~0.16 eV< Δ). They exhibit an analogous temperature behavior across the IMT. This behavior is better shown in Supplementary Fig. 7**d**. where the normalized increment of σ_1 ($\Delta\sigma_1 = (\sigma_1(180\ K) - \sigma_1(77\ K))/\sigma_1(180K)$) is plotted at both frequencies.

A further reason of our choice for the probe frequency is related to the temporal resolution as the manuscript is mainly focused on the dynamics at short timescale which could not properly resolved using a THz probe pulse. In fact, the state-of-art of THz generation allows getting quasi-single cycle THz pulses with a spectrum confined mainly between 0.5 THz and 5 THz, which roughly correspond to a pulse temporal duration on the order of half picosecond. For instance, in Supplementary Fig. 8 we report the intensity temporal profile of a THz pulse generated by a DSTMS crystal. As one can see, the pulse duration is 330 fs (FWHM). The probe a 0.67 eV proposed in the experiment has a temporal duration (FWMH) of 58 fs which can properly solved the ultrafast metallization dynamics induced by the THz pump.

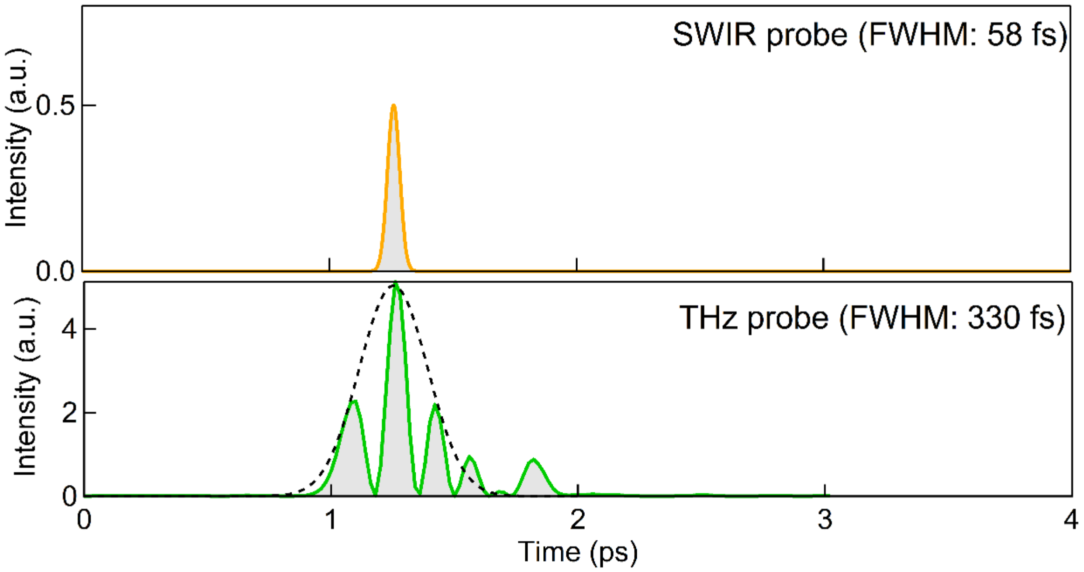

Supplementary Fig. 8. Temporal profile of the intensity of SWIR probe and for THz probe. Dashed line corresponds to a Gaussian envelope fit. THz probe pulse was generated by a DSTM crystal. (Full width half maximum from Gaussian fit: $FWHM_{SWIR} = 58\ fs, FWHM_{THz} = 330\ fs$).

In Supplementary Fig. 9**a-b**, we compare THz pump-probe results at different probe photon energies (THz probe: 0.014 eV, SWIR probe: 0.67 eV and NIR probe: 1.55 eV). As one can observe, unlike the THz probe and the SWIR probe, the modulation of the NIR probe is very small (less than 5%), indicating that NIR probe (which is above the V_2O_3 optical gap) is not very sensitive to the metallic response.

In Supplementary Fig. 9**d**, we report the estimated optical conductivity, by the chance in transmission using the Tinkham formula[3], at different pump probe delay (t_{pp}) compared with the optical conductivity at 4 K.

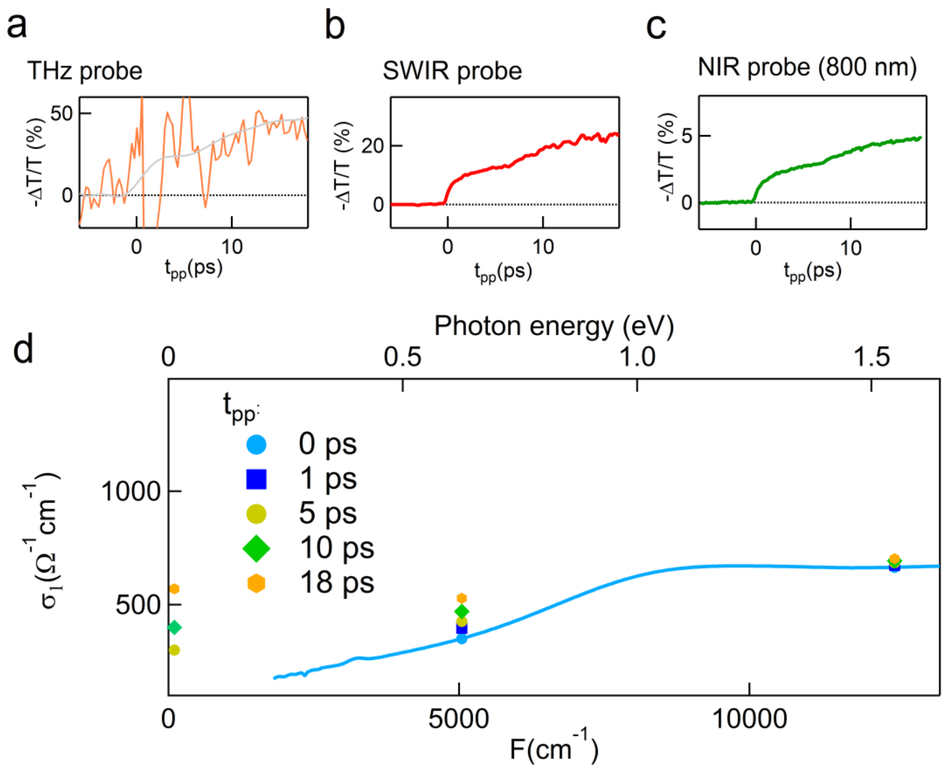

Supplementary Fig. 9. a-c, Time resolved THz induced transmission modulation $-\Delta T/T$ in the THz range, SWIR range (1850 nm) and NIR range (800 nm) at 4 K. THz probe was generated by a DSTMS crystal (Supplementary Fig. 8). The gray curve in panel **a** is a guide to the eye. **d,** Related optical conductivity at different pump-probe delay (t_{pp}). Optical conductivity computed by the change in transmission using the Tinkham formula. The blue curve is the insulating phase measured by FTIR spectroscopy.

Supplementary Note 7. Ultrafast IMT: THz pulse excitation Vs NIR pulse photoexcitation

To better clarify the picture of the high field THz excitation in V_2O_3, it is very important to compare the THz electric-field induced Mott transition with those generated by photo-irradiation. For this purpose, we provide further data performing near infrared (NIR) pump - SWIR probe spectroscopy of V_2O_3 at 4 K as measured by the THz pumping.

In Fig. R1a and b, we report the dynamics of the transmittance modulation $-\Delta T/T$ at 4 K vs. the delay time among pump and probe t_{pp} driven by the THz and near-infrared pulses, respectively. Both $-\Delta T/T$ show a similar variation vs. time.

The yellow line in Supplementary Fig. 10**a** and the purple line in Supplementary Fig. 10**b** are the intensity profile of the THz $(E_{THz})^2$ and the NIR pulses INIR, respectively. The experimental conditions of the THz pulse excitation are those specified in the main manuscript. For the NIR

experiment, the pump photon energy was centered at 1.55 eV (800 nm) and the pump fluence was set at 3 mJcm^{-2} as is Ref. 10. The NIR pump pulse has a Gaussian temporal profile, $\exp(-t^2/\delta^2)$ with δ=34 fs, as measured by a SRSI (Wizzler-Fastlite).

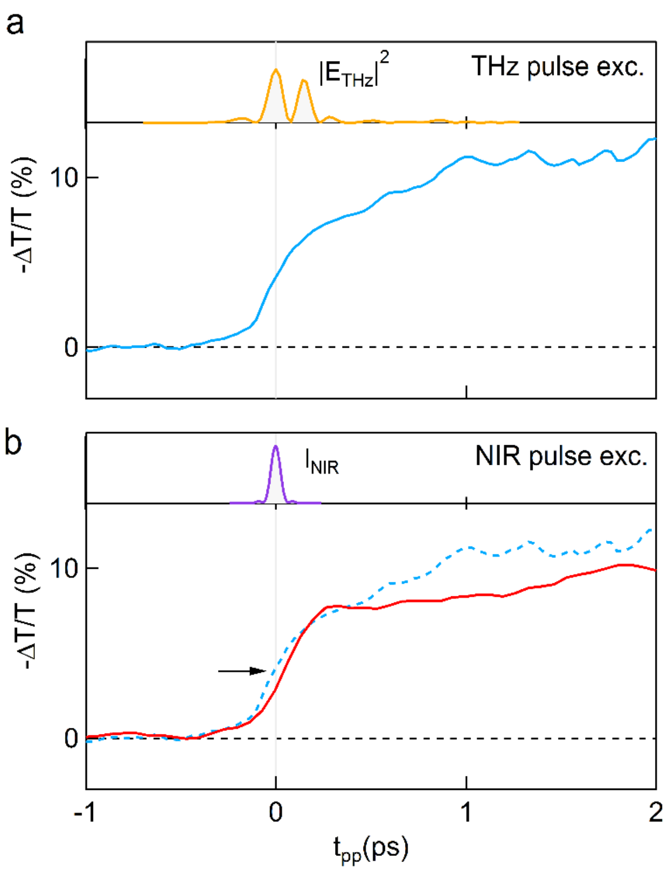

Supplementary Fig. 10: Comparison between terahertz pump excitation and NIR pulse excitation dynamics of V_2O_3 at 4 K. a) temporal dynamics of terahertz induced transmission change –**Δ**T/T at probe wavelength (1850 nm) as described in the main manuscript. **b**) Temporal dynamics of –**Δ**T/T at 1850 nm probe wavelength induced by a NIR pulse excitation (Fluence: 3 mJcm^{-2}). The NIR pump and the SWIR probe spot size (1/e^2 widths) are 1100 µm and 80 µm, respectively. The black dotted line in **b)** shows the –**Δ**T/T driven by THz pulse for comparison.

At lower temperature (4 K), the NIR excitation (which photon energy is higher than the optical gap) induces photocarriers leading to a fast transient photoconductivity as conventionally observed in band insulators[14].
The delay time of 55 fs at zero crossing in the pump-probe traces (black arrow) between the THz and NIR pump may be relevant but it cannot be well evaluated because it depends on the time-zero definition of the THz wave packet.

Supplementary Note 8. Transmission modulation Vs Temperature and THz electric field strength

In Supplementary Fig. 11**a** we report the behavior of -ΔT/T at t_{pp}=6 ps as a function of the THz electric field strength and temperature. As one can observe in the contour plot (Supplementary Fig. 11**a**), between 100 and 145 K, where the dynamics shows a thermal regime (see main manuscript), the increment of -ΔT/T with the THz electric field strength is anticipated and the electric field threshold tends to be lower. These behaviors are better shown in Supplementary Fig. 11**b**, where the

temperature evolution of -ΔT/T is reported for three different electric field strengths (8.0, 3.8 and 0.5 $MVcm^{-1}$). This temperature behavior can be related at two effects: 1) the presence of conductive states, as describe in the main manuscript, which directly interact with the THz pulses allow a more efficient absorption of energy (presence of leakage current). 2) The lattice temperature is closer to the transition temperatureϑ_{IMT}, which means that a minor density of energy is required for the thermal insulator to metal transition.

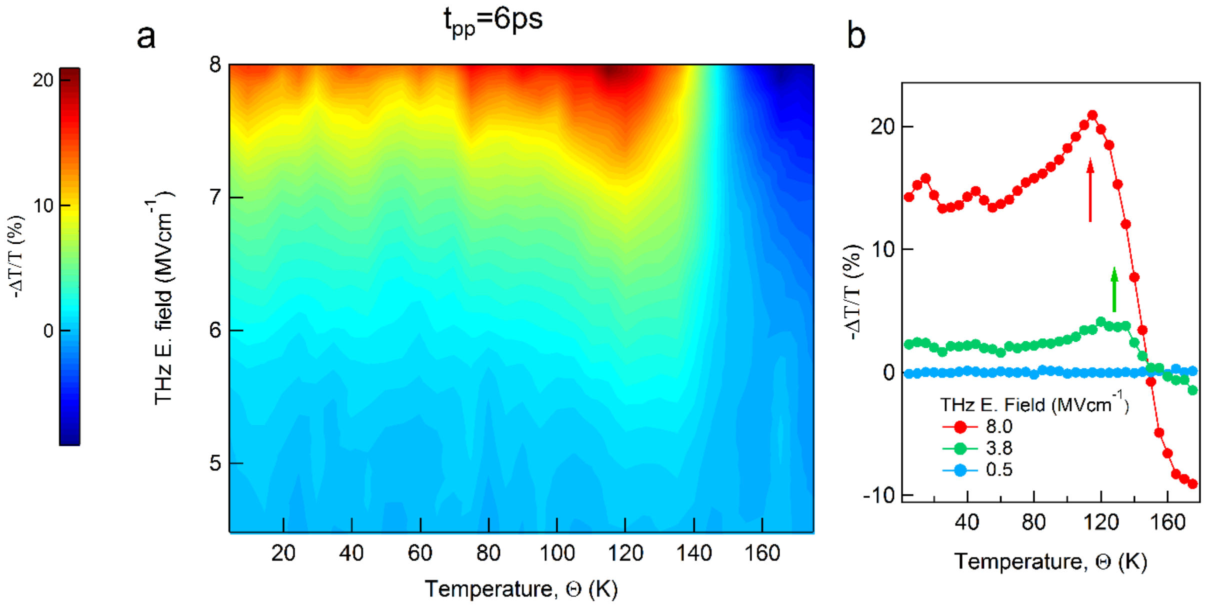

Supplementary Fig. 11. THz electric field dependence of -ΔT/T at t_{pp}=6 ps as a function of temperature. **a**, SWIR transmission modulation –ΔT/T vs electric field strength and temperature. **b**, temperature evolution of –ΔT/T at 8.0, 3.8 and 0.5 $MVcm^{-1}$ of electric field strength. Arrows indicate the maximum of -ΔT/T.

Supplementary References

1. Vicario, C., Monoszlai, B. & Hauri, C. P. GV/m single-cycle terahertz fields from a laser-driven large-size partitioned organic crystal. *Physical Review B* **112**, 213901 (2014).
2. Roskos, H. G., *et al*. Broadband THz emission from gas plasmas induced by femtosecond optical pulses: From fundamentals to applications. Laser & Photonics Reviews **1**, 349-368 (2007).
3. Henning, P. F. *et al*. Infrared studies of the onset of conductivity in ultrathin Pb films. *Physical Review Letters* **83**, 4880 (1999).
4. Glover III, R. E., & Tinkham, M. Conductivity of Superconducting Films for Photon Energies between 0.3 and 40kT_c. *Physical Review Letters* **108**, 243 (1957).
5. Qazilbash, M. M. *et al.* Electrodynamics of the vanadium oxides VO_2 and V_2O_3. *Physical Review B* **77**, 115121 (2008).
6. Lamsal C. & Ravindra N. M. Optical properties of vanadium oxides-an analysis. *Journal of materials science* **48**, 6341-6351 (2013).
7. Kuzmenko, A. B. Kramers–Kronig constrained variational analysis of optical spectra. *Review of scientific instruments* **76**, 083108 (2005).
8. Liu, M. K. *et al.* Photoinduced phase transitions by time-resolved far-infrared spectroscopy in V_2O_3. Phys. Rev. Lett. **107**, 066403 (2011).
9. Lo Vecchio, I. *et al.* Orbital dependent coherence temperature and optical anisotropy of V_2O_3 quasiparticles. Journal of Physics: Condensed Matter **29**, 345602 (2017).
10. Abreu, E. *et al.* Ultrafast electron-lattice coupling dynamics in VO_2 and V_2O_3 thin films. *Physical Review B* **96**, 094309 (2017).
11. Baldassarre, L., et al. Quasiparticle evolution and pseudogap formation in V_2O_3: An infrared spectroscopy study. *Physical Review B* **77**, 113107 (2008).

12. Ronchi, A. *et al.* Nanoscale dynamics across the Mott transition in V_2O_3. Preprint at https://arXiv.org/abs/1807.03670 (2018).
13. Lo Vecchio, I. *et al.* Optical properties of V_2O_3 in its whole phase diagram. *Physical Review B* **91**, 155133 (2015).
14. Beard, M. C. *et al.* Subpicosecond carrier dynamics in low-temperature grown GaAs as measured by time-resolved terahertz spectroscopy. *Journal of Applied Physics* **90**, 5915 (2001).
